# Testing the limits of large language models in debating humans

**DOI:** 10.1038/s41598-025-98378-1

**Published:** 2025-04-22

**Authors:** James Flamino, Mohammed Shahid Modi, Boleslaw K. Szymanski, Brendan Cross, Colton Mikolajczyk

**Affiliations:** 1 Department of Computer Science and Network Science and Technology Center, Rensselaer Polytechnic Institute, Troy, NY USA; 2 Department of Mathematics, Troy, NY USA

**Keywords:** Large language models, Artificial intelligence, Human–computer interaction, Social dynamics, Opinion consensus, Statistical analysis, Computer science, Statistics, Scientific data

## Abstract

Large Language Models (LLMs) have shown remarkable promise in communicating with humans. Their potential use as artificial partners with humans in sociological experiments involving conversation is an exciting prospect. But how viable is it? Here, we rigorously test the limits of agents that debate using LLMs in a preregistered study that runs multiple debate-based opinion consensus games. Each game starts with six humans, six agents, or three humans and three agents. We found that agents can blend in and concentrate on a debate’s topic better than humans, improving the productivity of all players. Yet, humans perceive agents as less convincing and confident than other humans, and several behavioral metrics of humans and agents we collected deviate measurably from each other. We observed that agents are already decent debaters, but their behavior generates a pattern distinctly different from the human-generated data.

## Introduction

The advent of AI has given rise to a dream of building artificial agents capable of partnering and even successfully competing with humans^1^. A baseline of successful competition between AI and humans was accomplished in 1997 when IBM’s Deep Blue defeated Garry Kasparov, the World Champion at the time^2^. Later, a notable milestone was set in 2011 with IBM’s Watson, which exhibited a robust understanding of human language and defeated human champions in *Jeopardy!*^3^. In 2022, a breakthrough occurred in AI-human dialogue with the Introduction of Large Language Models (LLMs) such as ChatGPT^4^. These powerful models, trained on a vast number of human-produced texts, can converse with humans and solve complex text-based tasks^5–9^. Their ability to realistically interact with people using human language in a contextually and semantically cohesive fashion could transform social science, as postulated in^10^ wherein the authors discuss scenarios in which LLMs could act as confederates to facilitate human-based experiments or even as surrogates for human players. Both roles have received some attention from designers of LLMs^6,11,12^. Although some past work has explored the role of artificial confederates in behavioral experiments^13^, those confederates acted in a limited role. They lacked the depth of reasoning that LLMs possess.

Testing if LLMs can behave like human confederates is crucial, as these models potentially pose an unprecedented opportunity for researchers to break the traditional mold of human-based experimental design. Today, little is known about the behavioral dynamics of human-LLM conversation, notably in a study setting. Subsequently, in this paper, we describe an experiment that robustly and rigorously tests the limits of agents in acting as confederates or when surrogating humans.

Our paper uses the term ’agents’ to describe the simulated players that participate alongside human players in our debate games. These agents generate conversational messages using a combination of two LLMs, GPT-4 and Llama 2. They use ChatGPT with custom prompts and Python scripts to interact with the game environment and maintain a memory of past conversations within a single game. Thus, any agents with the same persona have no memories of past games, so each game is the first for each agent and human. The agents are assigned unique ’personas’ to individuate them from each other as separate entities. The ’personas’ are a unique combination of four initial traits: stubbornness, grammar sophistication, personal confidence and personally preferred diet. Any agent with the same persona in two or more separate games is considered the same individual in those games. (See “Agent Design” in the Methods section for details on each personality trait and the capabilities of agents.)

Within each game, agents, like humans, maintain memories of interactions with other players and could refer to previous discussions in follow-up conversations with players they had already spoken to. Agents did not share memories and were, like humans, unaware that other agents could be present in a game. Each agent cleared memories of the previous games at the beginning of a new game. Thus, each game was the first played from the agent’s point of view.

We seek to answer two questions relating to the capabilities of these agents in conversation-intensive studies: How capable are agents of acting as confederates (i.e., preserving or enhancing opinion dynamics leading to consensus), and how capable are they of being human surrogates, generating data indistinguishable from human data in the same environment?

Considering the implications of these questions, we look to approach this research through the prism of social science. First, we determined the number of samples necessary to achieve statistical significance of our debate experiment’s results^14,15^. We identified all the required measures before initiating the study. We then pre-registered the research and implemented a battery of statistical tests (including Bayesian regression models^16^) on the data collected post-experiment.

The debate experiments we ran consisted of multiple games, starting with six players that involved a combination of humans and agents acting as humans, all anonymously. We had three types of games with seats for either six agents, or six humans, or three agents and three humans, denoted respectively as *AA*, *HH*, and *AH* games. The humans were informed that agents were engaged at the end of the game. The game aim is to convince other players of your opinion on a debate topic and to seek a majority consensus. For our study, the debate topic was on the best diet choice. Specifically, players were given this prompt at the beginning of a game, verbatim: “Which of these diets is the best compromise between nutritiousness and climate consciousness?” players were then asked to select one of these four choices of diets: Vegan, Vegetarian, Omnivorous, or Pescatarian. We chose this topic for the prompt because a choice in the diet affects a wide range of issues (including environmental and societal) but does not trigger deep societal polarization intrinsic to topics like politics, which often produce opinion stagnation^17^.

To promote collaboration among players, we designed a reward point system: convincing another player to change their opinion grants the persuader one point, and all players with the most popular opinion at the end of the game receive three additional points. At the end of the game, the top two players are awarded double their base compensation. The players are given one hour to debate one-on-one with all other players using a custom texting interface. These rules are revealed to players before the game. The game is intentionally simple in its design, with the sole source of interactions being pairwise, anonymous, open-ended text-based messaging sessions, enabling us to collect all text exchanges, dynamics of opinions about diets, and temporal aspects of interactions.

Recording the resultant behavioral patterns and comparing them across game types gives us the data to answer our two questions. In the following sections, we describe our experiment and the data we collected, and then we begin analyzing our results and comparing differing behaviors across varied study players.

## Experiment design

We deployed our study using voluntary players from the student body of the Rensselaer Polytechnic Institute. A total of 251 students signed up for the study; 111 humans joined games, but only $N_h=97$ actively participated in conversations. This human persona attrition led to some AH and HH games having fewer than three or six humans, respectively. We decided to include games with partial populations in the analysis of results since we found that they improve the accuracy of these analyses without skewing the results (see SM section 7, 7.1 and SM table S8). We organized and ran to completion 37 games, of which $N_{HH}=10$ were *HH* games, $N_{AH}=17$ were *AH* games, and $N_{AA}=10$ were *AA* games. See “Agent Design” in the Methods section for the detailed experimental procedure and explaining how we scheduled games and decided human-agent player groupings.

For the 17 *AH* games, we generated 30 agents with unique personas. Some agents with the same personas were present in more than one *AH* game. For the 10 *AA* games with six agent seats each, we generated 60 agents with unique personas. Thus, we had a total of 90 agents with 90 unique personas, and used $N_a = 90$ as the population size of agents. When analyzing the behavior of agents in the results section, we treat the persona of the agents as an individual subject, employing repeated measures analysis to account for the multiple data points submitted by these subjects. All agents starting a new game are cleared of any memories of the previous games, so all humans and agents play each game for the first time.

A sample size analysis indicated that to achieve a medium effect size in our comparisons, we needed at least 10 games per type; see the Supplemental Materials (SM) Section 7 for details. We note that for *AH* games, we set the number of games to 17 to increase the data on human-agent interaction. Player assignment to condition was random; whenever the time slot for a game registered six humans, we would play an *HH* game until we had ten games played. With more than two registered humans, we randomly selected three human players and generated three random agents for an *AH* game. For *AA* games, we generated six random agents.

**Fig. 1:**
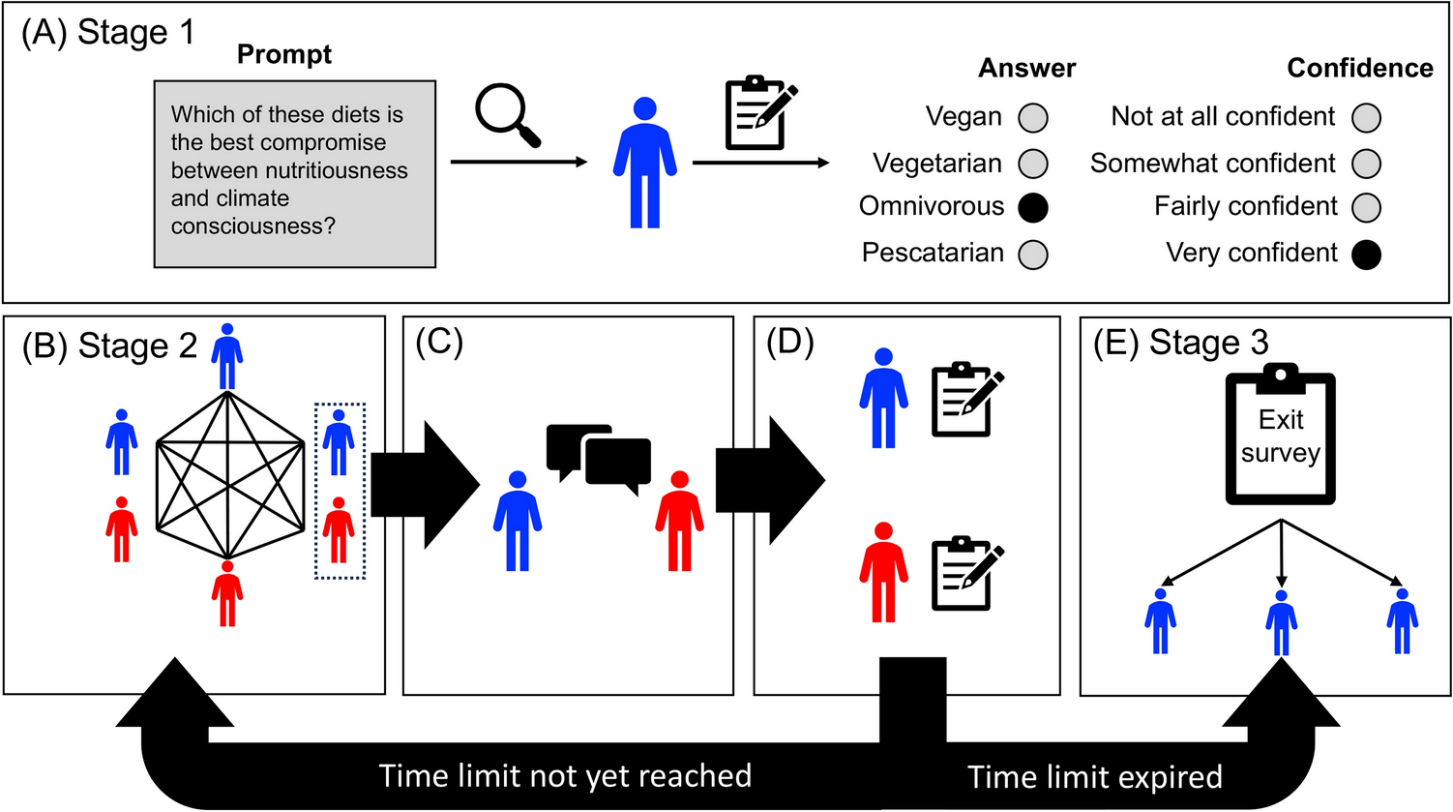
Experimental setup for a *AH* game type. (**A**) Stage 1: The players select an initial opinion and rate their confidence. (**B**) Stage 2: The players are placed in a fully connected network with five other players who completed stage 1. The blue icons represent human players in this network, and red represent agents. The players can then invite each other to engage in one-on-one conversations. (**C**) After two players agree to converse, they are cut from the network and converse using a text-based messaging system. (**D**) Once the conversation terminates, both players re-evaluate their opinions and personal confidence and assign a level of perceived confidence to their conversational partner. Then they rejoin the network (see **B**). If the time limit has expired, all active players move to stage 3. (**E**). Stage 3: The game ends, human players file an exit survey, and all players quit the study.

Figure 1 depicts our game design. In stage 1 of each game (Fig. 1A), each player is given the prompt, which lists four opinion choices from which each player chooses an opinion and rates their confidence in their selection on a four-level scale: “not very confident,” “somewhat confident,” “quite confident,” and “very confident.” In stage 2, each player is assigned to a fully connected network of size six. (Fig. 1B). Each player in stage 2 can request a one-on-one conversation with any other player in their network. If their request is accepted, those two players temporarily exit the network and engage in a private conversation via text-based messaging. The conversation lasts until one of the conversational partners terminates it or the time limit expires (Fig. 1C). When a conversation ends, both players can re-evaluate their opinion and their level of personal confidence. They are also asked to rate their partner’s perceived confidence using one of the same choices as personal confidence, with an additional “not enough info” option. After completing this step, time permitting, both players are returned to the network, and each can request or accept subsequent conversations from other players (Fig. 1D).

Once the time limit has expired, all active players move to stage 3 (Fig. 1E), in which human players file exit surveys (see “Exit survey design” in the Methods for more details). Then, all players of this game are removed from the game and the study.

Our final dataset includes 713 conversations, of which 591 were completed before the time limit expired.15,804 messages were sent in those conversations, resulting in 238 opinion changes. We also collected 91 exit surveys from the human players in the *AA* and *AH* games. Of all the conversations made in the *AH* games, 152 were between a human and an agent, 30 between two humans, and 82 between two agents, respectively. In the *HH* games, 140 conversations were between two humans, while in the *AA* games, 309 conversations were between two agents. Excerpts of conversations occurring in three game types are shown in SM Table S24.

## Results

We focus on three behavioral metrics for each player. The first metric is the fraction of conversations that resulted in opinion change. The second is the frequency of changing all self-reported personal and perceived confidences. Our final measurements focus on productivity, which applies metrics of player outputs based on statistical data collected in-game and synthesized post-game. The former involves the number of conversations, their message counts, and the count of the points awarded to players at the end of the games. The latter focuses on conversational attributes, including the fraction of on-topic messages sent during conversations. Both in-game and post-game productivity measures enabled us to detect when humans and agents behave differently comparatively.

With two player types (human and agent) and three game types (*AA*, *AH*, and *HH*), there is a variety of data that can be grouped differently. Subsequently, we classify conversations into three categories: *aa*, *ah*, and *hh*, indicating conversations between two agents, an agent and a human, and two humans, respectively. The homogeneous games can only generate corresponding homogeneous discussions (e.g., *HH* games create *hh* conversations). However, *AH* games can produce all conversation types. When reporting on conversations in *AH* games that involve opinion change, we indicate which type of players changed their opinion by changing the order of the a and h, where the first letter is the indicator. Hence, the behavior of humans in AH games. Each AH conversation generates two data points, one for *ah* and one for *ha*.

A similar distinction is needed when players assign a level of perceived confidence to their conversational partner. As such, we draw an arrow from the assigning player type to receiving player type: *a*$\rightarrow$*a*, *a*$\rightarrow$*h*, *h*$\rightarrow$*a*, *h*$\rightarrow$*h*. These indicate the assignment of perceived confidence from an agent to an agent, an agent to a human, a human to an agent, and a human to a human, respectively. We refer to this notation as “assignment type.”

### Dynamics of opinion switching

In consensus games, success is to convince another player to adopt one’s opinion. We analyzed how often human players change their opinions when conversing with other humans or agents. We measured the opinion change frequency of players using a Bayesian multilevel logistic regression model predicting the probabilities of such changes as a function of the player and their conversational partner types. See Table 1 for all instances of post-conversation opinion changes across the three types of games, clustered by conversation type.

**Table 1:** Summary of conversations and opinion change in all games.

Game Type	Conversation type	Did the player change opinion?	
		Yes	No
*HH*	*hh*	32	218
*AA*	*aa*	114	458
*AH*	*hh*	7	38
	*ha*	4	130
	*ah*	43	91
	*aa*	38	110
Total		238	1046

This table contains the opinion change outcome of all 642 completed conversations across all three game types. Conversation type indicates the type of user interaction: two humans (*hh*), two agents (*aa*), or an agent and a human (*ah*). For *ah* conversations, we show two rows where the first letter indicates for which player, agent, or human we measure opinion change. Each player in a conversation has the opportunity to change their opinion, yielding $642 \times 2$ opinion changes. We note that incomplete conversations do not record any opinion changes, and there were cases in which one player finished re-evaluating their opinion before the time limit expired, but their conversational partner did not. See SM Table S22 for a version of this table further split by the type of player who initiated the conversation.

Table 2 summarizes the results of our Bayesian model, shown as the odds of opinion change for humans and agents in each conversation type. The odds ratio compares the odds of opinion change for each conversation type against the human-human (*hh*) conversations, for example $\text {Odds-Ratio}_{ha} = {\text {Odds}}_{ha}/{\text {Odds}}_{hh}$. The 95% credible intervals (CI) shown are in Odds of opinion change. For more details on the regression model, including formula and raw coefficients, see SM Section 9 and SM Table S11.

**Table 2:** Bayesian multilevel opinion changing model.

Conversation type	Odds ratio	Odds	CI (95%)
*hh*	1	0.12	0.068–0.185
*ha*	0.16	0.02	0.003–0.046
*ah*	2.99	0.36	0.203–0.574
*aa*	1.97	0.23	0.162–0.319

Observations	1284		
$N_{game}$	37		
$N_{player}$	185		
$\sigma ^2$	3.29		
$\tau _{00\ game}$	0.10		
$\tau _{00\ player}$	0.76		
Marginal $R^2$	0.030		
Conditional $R^2$	0.140		

This table outlines the results of our regression model, presented as the odds of opinion transitions in conversations and odds ratios relative to *hh* conversations. Note that in *ah* and *ha* conversation types, the first letter indicates the player type, which changes opinion. This analysis only includes data from completed conversations. Thus, this analysis excludes the 14 players who didn’t actively participate in their respective games and the two who conversed for the entire game time without closing the conversation.

**Fig. 2:**
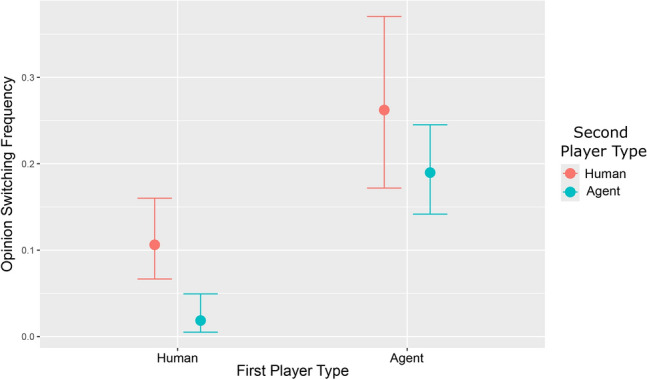
Bayesian multilevel model for opinion changing by player type. This plot shows the frequency of opinion changes as a function of the two conversing players. Each player in a conversation is a human or an agent, and combining these two types gives us an opinion-switching frequency for all conversation types in the study.

Figure 2 shows the mean and credible interval of the opinion-switching frequency for each type of conversation that could occur in the study. In *hh* conversations, the expectation is that 0.12 players will switch their opinion for every non-changing player mean odds: 0.12, $95\%$ CI 0.07–0.19). The *ha* odds ratio (mean value 0.16, $95\%$ CI $0.04 - 0.47$) shows that for every opinion switched in *hh* conversations only 0.16 opinions switch for *ha* conversations. The inverse of this, 6.25, shows that humans switch their opinions 6.25 times more often when interacting with other humans vs agents. Therefore, on average, agents need 6.25 times more conversations with humans to induce the same number of opinion switches that *hh* conversations induce. We also found that the rate of opinion change for *ah* conversations can depend on what type of player holds the opinion proposed for switching to. For more details on this finding, see SM Section 13 and SM Tables S20, S21, and S22.

Overall, the above results imply for question 1 that agents are far less convincing for humans than humans. Relevant to question 2, the differences in the frequencies of opinion switching seen between agents and humans are confirmed by our regression model in Table 2. When we average over levels of the second player type, we get that humans change opinion with an odds of 0.047 ($95\%$ CI 0.023–0.083), while agents change opinion with an odds of 0.288 ($95\%$ CI 0.201–0.408). This means agents, on average, have a 6.1 times higher odds of switching their opinion when compared to human participants.

### Personal and perceived confidence

**Fig. 3:**
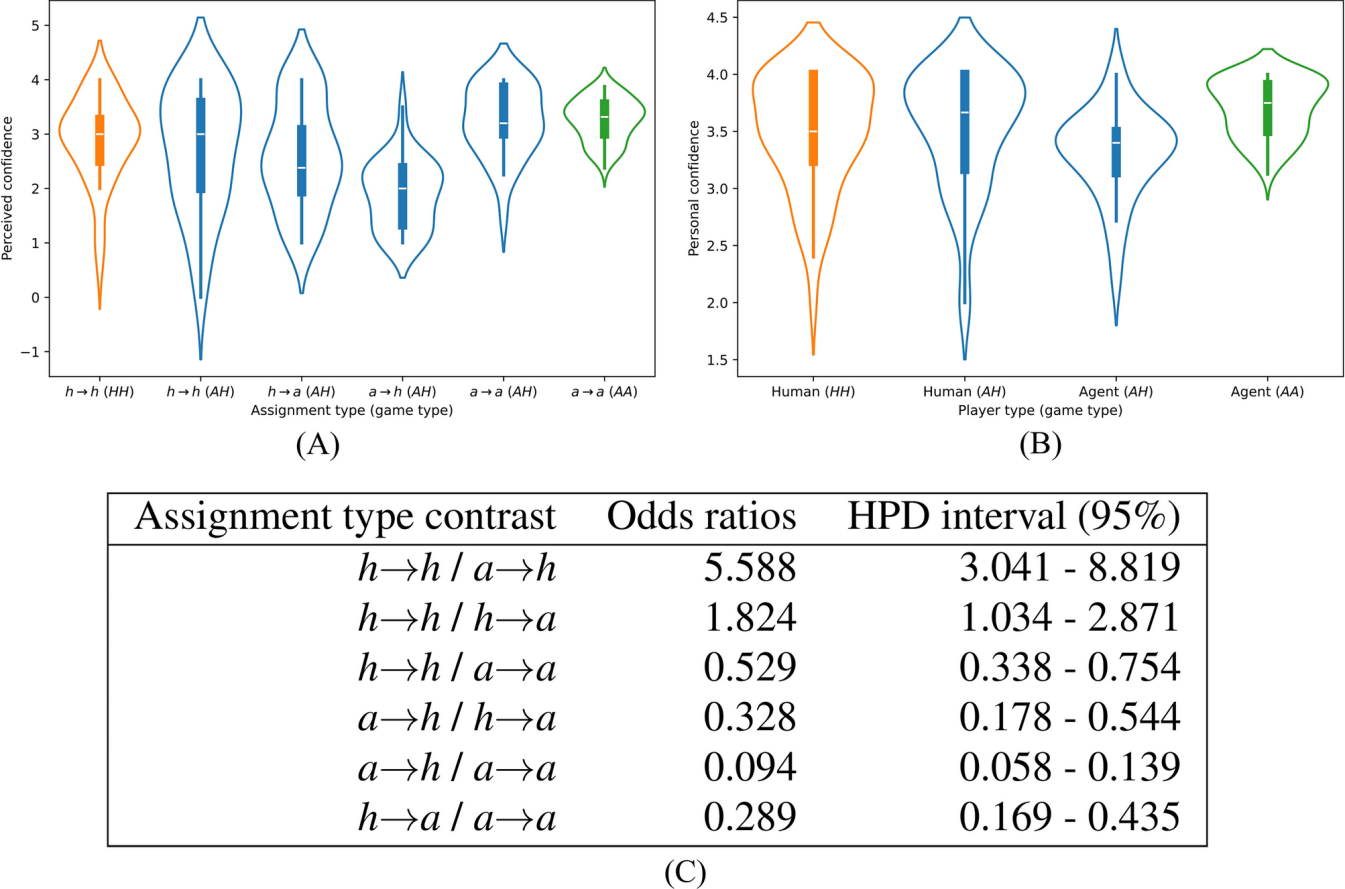
Distributions and modeled assignment type contrast for perceived and personal confidences. (**A**) This shows violin plots of perceived confidence, clustered by type of assignment and of game (indicated in parentheses). (**B**) This shows violin plots for personal confidence, clustered by player type and game type (also indicated in parentheses). (**C**) The embedded table shows the contrasts between assignment types in the perceived confidence Bayesian regression model. Estimates are given as odds ratios between contrasting conversation types. The highest posterior density (HPD) interval defines the shortest interval containing 95% of the posterior mass for the given estimates.

Figure 3 displays the distributions of confidence exhibited by players by type of game and assignment for both perceived and personal confidence. We numerically encoded the confidence levels, from the “Not very confident” level assigned as 1 to “Very confident” assigned as 4. Additionally, “Not enough info” was assigned 0 for perceived confidence. In Fig. 3A, we clustered perceived confidence levels by their assignment type, while for Fig. 3B, we clustered personal confidence levels by player type.

We again use a Bayesian multilevel model to compare the distributions of perceived confidence from *h*$\rightarrow$*h* and *h*$\rightarrow$*a*. The model reveals a statistically significant difference in how perceived confidence is assigned. Furthermore, the model suggests a substantial difference in the perceived confidence being assigned by humans to agents compared to agents to humans. The complete model specifications are shown in SM Section 9 and SM Table S12.

In terms of results, Fig. 3(C) shows the assignment type contrasts in perceived confidence assignment for the perceived confidence model. The model establishes that humans perceive other humans as more confident than agents by mean value 1.8, $95\%$ HPD-interval 1.034–2.871). Conversely, agents assign a significantly lower level of perceived confidence when interacting with humans compared to other agents by a factor of 10 (mean value 0.094, $95\%$ HPD-interval $0.058 - 0.139$). These differences in perceived confidence between humans and agents are statistically significant for most assignment types.

We extend this analysis to personal confidence as well. Implementing a Bayesian Multilevel model to predict personal confidence as a function of conversation type, we found that no statistically significant relation exists between conversation type and the players’ confidence (see SM Table S13). Concerning question 1, the above analysis indicates that agents negatively affect a human’s perceived confidence but do not affect personal confidence.

The above results have implications for question 2 as well. Notably, agents consistently perceive other agents as more confident, and humans tend to treat other humans likewise. The consistency of this behavior identifies a degree of similarity in the two-player types in terms of assigning perceived confidence to the same player type.

### Post-game productivity

**Fig. 4:**
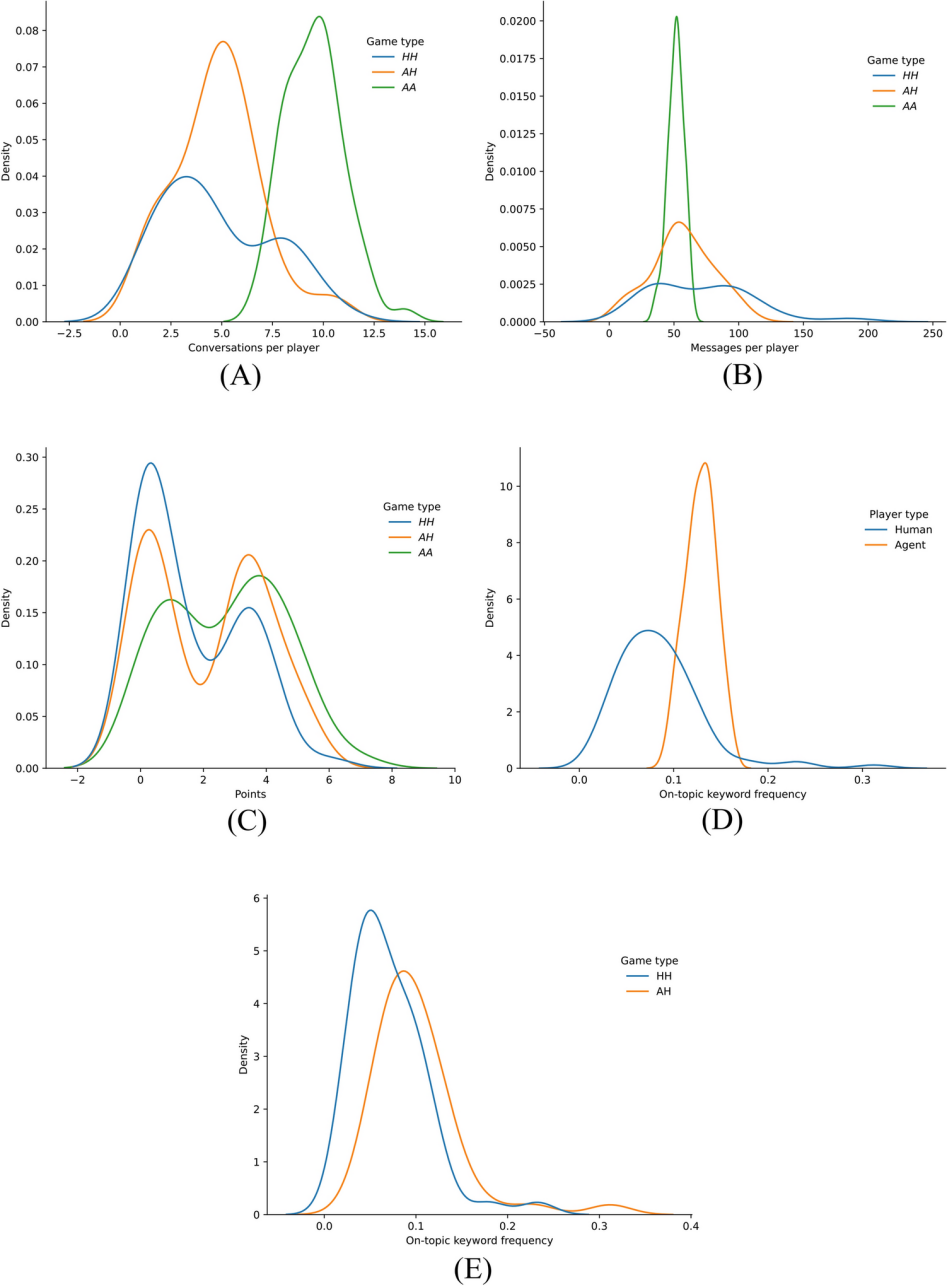
Post-game and in-game productivity results. (**A**) Kernel Density Estimations (KDEs) of the number of conversations per player grouped by the type of game they played in. (**B**) KDEs of the number of messages each player sent within each game, grouped by their type. (**C**) KDEs of the reward point distributions gained by all players at the end of each game, grouped by their type. (**D**) KDEs of the on-topic keyword frequency of each player, agent and human, across all game types. (**E**) KDEs of the on-topic keyword frequency of each human in *AH* game versus humans in *HH* games.

Next, we analyzed changes in productivity. The first three sub-figures of Fig. 4 show the results of post-game analyses on the number of conversations and message counts (Fig. 4A, B) and point distribution (Fig. 4C).These measures are aggregated over each player (human participant and agent persona) such that each player provides one data point representing their average behavior throughout the study. For the conversation and message counts, our visualizations make apparent a spectrum of behavior in *HH* games and *AA* games. Conversations and messages from *AA* games tend to exhibit distributions with a tighter modality, indicating that agents’ communication behavior is much more static. This makes sense, as the agents were designed with a message budget: a maximum number of messages allowed to be sent before the conversation is terminated. See “Agent Design” in the Methods for details on the implementation. We also further explore the relationship between the number of messages sent per conversation and the influence of the message budget in SM Section 8.

Compared to the communication behavior seen in *AA* games, *HH* communication varies much more, as illustrated in the wider modalities of Fig. 4A, B. The *AH* games show a mediation of these two extremes, indicating the presence of agents in these games adds a level of predictability. In the context of question 1, we found that the more agents narrow their variances of conversation length and count, the more humans conversing with these agents narrow their corresponding variances too.

Agents also facilitated the distribution of award points in games, earning more points overall than humans (Fig. 4C). This suggests that agents were likelier to stay focused and attempt to promote opinion change in themselves and others. This observation is essential for question 2, indicating that agents are more goal-oriented in this setting. We report majority consensus groups for *AH* games in SM section 15 and SM Table S23.

### In-game productivity

The second subcategory in our productivity analysis relates to in-game productivity, like on-topic keyword usage. Here, we define on-topic keyword usage as the number of occurrences of specific words that we identified as related to the game’s prompt (i.e., diets, nutrition, climate) or opinion consensus (i.e., team, majority, minority). More information on the dictionary of keywords used can be found in “Keyword analysis” in the Methods.

Fig. 4D shows KDEs of the frequency of on-topic keywords for each player involved in the study, grouped by their player type (i.e., an agent or a human). Each player provided one data point representing their frequency of using on-topic keywords across all conversations they participated in. One primary observation from this data is that agents generate more on-topic keywords than humans. The mean and standard deviation of the human distribution from Fig. 4D are 0.085 and 0.046, respectively, compared to 0.129 and 0.016 for the agent distributions.

Figure 4E presents a similar analysis to Fig. 4D, except we only consider human player on-topic keyword frequencies and group them by the type of game they participated in. Importantly, we find that agents’ proclivity for on-topic discussion affects the behavior of humans that are in the same game, as indicated by humans in *AH* games generate more on-topic keywords than humans in *HH* games ($p=$ 0.0018, Welch’s t-test^18^). We extend this analysis to unique word counts in SM Section 10. Additionally, we further probe the dynamics of player behavior during conversation by analyzing messages’ response times and holding periods, shown in SM Section 2 and SM Table S3. This analysis reinforces that agent presence affects humans during conversation, with agents often making humans slower to respond. We also probe changes in in-game productivity based on conversation initiator in SM Section 13 and SM Tables S17, S18, and S19.

Ultimately, these results shed further light on question 1, as agents can elevate human engagement. Furthermore, in answer to question 2, the results demonstrate that agent word usage during conversation is measurably different from humans.

### Exit survey results

In stage 3 of our game (Fig. 1E), all human players are asked to fill out an exit survey, in which they must nominate the players they thought were the most and least convincing. At the end of the study, for the *AH* games, 18 agents and 24 humans were nominated as the most convincing players. In contrast, 31 agents and 11 humans were nominated as the least believable. These results indicate that the human players who took the exit survey were likelier to nominate other humans than agents as most convincing ($p=0.0025$, Boschloo’s exact test).

### Agents’ ability to blend in

Considering the tenuous relationship forming between humans and AI in the face of the fast-paced integration of AI into everyday life^19,20^, we implemented an exploratory measure for the agents’ ability to blend in with the human players during the games. We kept track of the instances in which humans accused a player of being an agent, which we call an agent detection incident (ADI). To recognize ADIs, we implemented a keyword filter on all messages produced by humans in the *AH* game type. This filter flagged any messages where the words “bot”, “AI”, “ChatGPT”, or “chatbot” were mentioned. Within *AH* games, 14 human players identified an agent during the conversation. This is $27.4\%$ of the human player population in *AH* games, with these ADIs occurring across 9 of the games, leaving 8 *AH* games with no incidents. In the 9 *AH* games with ADIs, 13 of the human players did not identify any agents. Therefore, humans who detected an agent did not always spread that information to the other human players. Interestingly, we observed that agents failing to cover their identity during one conversation does not imply that they will continue to fail afterward. One false ADI was also in an *HH* game.

We found that not enough players were involved to statistically verify any change in human behavior in the presence of ADIs (see SM Section 4 and SM Tables S4, S5, S6, and S7 for more details). However, we probed for more subtle behavioral changes by analyzing conversational partner selection bias in *AH* games (see SM Section 11). This test established that humans did not prefer connecting to other humans over agents in-game, and the same behavior was also found in the agents. We also performed a thorough qualitative study of why agents were detected and their context. We analyzed every ADI from the *AH* games to see what agent and human behavior led to agent detection. We designated four “triggers of detection” categories where at least one can be assigned to each ADI case. These categories are *AI system provoked*, *AI language provoked*, *human provoked*, and *human-human spread*. The categorization methodology is described in “Agent detection categories” in the Methods section. There were 21 *ah* conversations and 7 *hh* conversations with ADIs. Of those 21 *ah* conversation ADIs, 13 were caused by AI system/language provoked detections (61.91%), and the other 8 had human “hunters” with pre-existing suspicion. Table 3 shows agent-provoked ADI causes. It follows that 13 of 21 humans in ADI *ah* conversations could have found agents organically and not from prior suspicion.

**Table 3:** *ah conversation ADI causes.* This table shows 13 *ah* conversations with Agent Detection Incidents that were system or language-provoked. The game number (first column) indicates the game where the conversation occurred, with some games having more than one conversation. Eight *ah* conversations with humans “hunting” for agents are not listed, and six *hh* conversations with human-human spread ADIs.

$\#$	Cause of ADI
1	System: Agent cut the conversations abruptly when their message budget is empty
2	System: Agent responded to itself
3	System: The agent greeted the user in the middle of the conversation multiple times
3	System: The agent greeted the user during the conversation
3	System: The agent got confused about the past conversation in the follow-up conversation
4	System: The agent responded to itself and argued against itself
4	Language: Agent repeated its phrasing too closely
5	Language: The agent was too lengthy, and there was a delay in its replies
4	Language: Agent repeated its phrasing too closely
3	Language: The agent was too lengthy and formal
1	Language: The agent was too lengthy and often triggered the not-on-topic flag
6	Language: The agent was too formal and repeated its Introduction later
7	Language: The agent was too lengthy and confident

## Discussion

In the Introduction, we established two questions we sought to answer with our study: how capable are agents in preserving or enhancing opinion dynamics leading to consensus, and whether agents can generate realistic, human-like debate data. By collecting data on human and agent interactions in opinion consensus games where the nature of the players is anonymous, our results shed some light on the behavioral differences between agents and humans and the nature of their interactions.

While it is important to note that our samples are not representative of the general population, it can be argued that the study’s population tests the limits of the AI agent’s ability to blend in, as the younger, more educated demographic of our research may be more capable of identifying patterns of AI behavior. As such, our results should be considered as part of stress-testing conversational AI capabilities instead of simply being a representative snapshot of expected behavior for humans in the US. In this context, our analysis can be considered more generalizable to the nascent and trending social media platforms^21^ where AI technology has or most certainly will be integrated.

In the rigorous analysis of our study’s data, we found that agents attempting to influence people to change their opinions are six times less likely to succeed than humans influencing other humans. This trend persists with perceived confidence assignment since humans perceived other humans as 1.8 times more confident than agents. Agents were found to switch opinions 6.1 times more often than human players. Agents also found other agents to be 10 times more confident than humans (Fig. 3C). Furthermore, agents produced more on-topic keywords when in conversation, even elevating the usage of on-topic keywords for humans that participated in games with them (Fig. 4).

An interesting observation from the above results is that agents’ productivity in initiating conversations and staying on-topic resulted in an increased number of points awarded among agents despite the agents’ less convincing and confident behavior. This outcome suggests that many agents initiated conversations and provided compelling arguments for their diets. This strategy is effective in a debate-based opinion consensus game.

In Summary, while our agents can increase the productivity of humans participating in an opinion consensus game, they hinder humans’ opinion-changing behavior and appear less confident in conversation. Subsequently, they must improve in this area to be capable confederates. Our agents’ underlying behavior fundamentally differs from humans’. They stay on-topic longer than humans. These differences in behavior set them apart from humans, so much so that we can use simple machine learning classifiers trained on a subset of the metrics discussed above to differentiate between *ah* and *hh* conversations and *AA* and *HH* games (see SM Section 12 and SM Tables S14, S15, and S16). This deviation in behavior means that our agents are not yet ready to become accurate human surrogates in producing behavioral data.

One limitation of our work is that it may not reflect how humans with prior knowledge of agent presence may behave in debates and conversations. Our goal was to investigate if agents can be effective confederates in such contexts. If we labeled agent players as agents from the beginning, humans may have approached them differently, even when sharing the same goal of reaching a consensus. We chose not to suggest anything about who the players are to avoid discounting agents’ arguments in the debate. Such a bias of humans would distort all measures of the agent’s capabilities to debate. We were also careful not to suggest that players are all humans by using colors to identify players instead of giving agents human names. The results indicate that initial good agent performance in the games in which a human player detected agents’ presence gave agents a chance to gain enough respect from humans that they stayed in the games. Moreover, the interrupted games were statistically similar to the uninterrupted games. Another limitation is the unknown generalizability of our results to AI generative models other than the ones we used. All current studies share this limitation since LLM models rapidly evolve. We expect that the initial uneasiness to accept agents some humans have will quickly dissipate as interactions with agents become ubiquitous, making our approach neutral to results.

We conclude that our agents show promise in conversing with humans but must evolve more before becoming capable surrogates or confederates in conversation-intensive sociological studies. Future work is essential, given that research on human-AI dynamics within sociology remains unexplored. Our paper creates a foundation for investigating interactions between humans and agents in a debate setting. In sociological studies, it establishes an archetype for exploring these or other LLM-based confederates. In future research, we plan to study the relationship between AI language more deeply, how humans discover AI in anonymous interactions, and semantic drift^22^.

## Methods

Due to the nature of the study, this research was reviewed and classified as exempt by the Rensselaer Polytechnic Institute (RPI) Integrated Institutional Review Boards (IRB). This decision is shown in IRB ID 2133 (approved on 5 September 2023). These files are available upon request of the corresponding author. Executing all methods, we applied the relevant guidelines and regulation. All players confirmed informed consent before entering the study. Our study’s experimental design and primary analyses were preregistered at https://aspredicted.org/6PX_5TD.

### Player recruitment

The human players in our study were recruited from the graduate and undergraduate student body of Rensselaer Polytechnic Institute across various departments (see SM Section 3 for a demographic breakdown). Recruitment efforts consisted of emails sent to students from department heads, supplemented by wanted ads hung on campus. All recruitment material directed students to join a Discord channel dedicated to this study. Upon joining the channel, they were provided the terms and conditions of the study, the game rules, and instructions on how to join a game. SM Section 14 details participant preparation for the game, their essential compensations, and additional awards.

The game is hosted on customized playable software on mobile and PC browsers. See SM Section 16 for screenshots of the study’s Discord channel and the game website, respectively.

### Exit survey design

Immediately after the 60-minute timer expires in the game, the human players are informed if they are one of the two winners, and then all are provided with a link to the exit survey. There are four different variants of exit surveys, two for each game type involving human players (*HH* and *AH*), with both having their own two variations: one for the winning players and one for the remaining players. There is no difference in the questions asked all survey variations. Still, slight differences in the auxiliary text shown on the surveys depend on the variation received.

All four versions include a message shown to players upon submitting the survey that informs them that this study has two human-involved game types: one with agents and one without. The survey specifies whether the player was playing with agents or just humans, depending on the type of game. Additionally, players who win the game are congratulated for winning the game within the survey.

The survey is divided into influence nominations, demographics, and payment information. For influence nominations, players are asked to subjectively identify which fellow players in their game were the most and least convincing. For this section, the players receive a list of usernames of the players involved, from which they can select one. The demographics are optional, and information about players’ age, gender, and ethnicity is collected. Payment information was required.

### Keyword analysis

We identified 102 on-topic keywords relating to the game prompt regarding diet, diet nutritiousness, and climate consciousness. Additionally, keywords relating to opinion consensus dynamics, such as majority or minority opinions, were added. An initial selection of 36 keywords was identified using the YAKE tool^23^. This was done by deploying the tool on a pool of all 15, 547 messages sent by humans and agents. To encapsulate all aspects of the prompt and game dynamics in our dictionary of on-topic keywords, 66 additional keywords were manually identified and added by authors. See SM Section 6 for a list of all the on-topic keywords used in this research.

### Agent design

The critical components of this study are the agents, as all analysis hinges on their ability to accurately represent the typical capabilities of the current-day LLMs we have chosen. We used a fusion of OpenAI’s ChatGPT and GPT-4^24^ and Meta’s Llama 2^25^ to power the agents. GPT-4 and Llama 2’s APIs are called for all conversational tasks, with ChatGPT’s API being used to process auxiliary requests.

Our game follows the long tradition of simulating players in games with social interactions as agents, often endowed with personalities (^26^^27^^28^). Upon the initialization of a game, agents are instantiated with unique personas. Each persona consists of four traits. Each trait is represented by a digit $d_i$. Thus, in our games, there are $p=4$ digits. Each digit has a single value in the trait from the range $[0,m_i-1]$. $d_1$ represents the level of stubbornness with three values (stubborn, regular, and suggestible) determining how strongly agents defend their favorite diet in conversations, hence $m_1=3$. $d_2$ stands for grammar sophistication levels with three values (lowercase, perfect, and reduced punctuation), so $m_2=3$. The third digit represents initial personal confidence levels with four values (not very confident, somewhat confident, quite confident, and very confident), which indicate the strength of belief of the agent in its own diet choice, so $m_3=4$. The fourth digit represents an initial favorite diet with four values, hence $m_4=4$. The number of unique codes is $\prod ^p_{i=1} m_i$.

Having four digits of personality $(d_1,d_2,d_3,d_4)$, we can compute the decimal code for personality as follows: let $M_1=1$ and for $1<i<p, M_i=M_{i-1}*m_{i-1}$. Then, the decimal code is $\sum _{i=1}^p d_i*M_i$. In our case $p=4$, and $M_2=3, M_3=9, M_4=36$. Therefore the decimal code (0, 0, 0, 0) is 0 and largest (2, 2, 3, 3) is $2*M_1+2*M_2+3*M_3+3*M_4=2+6+27+108=143$, so there are 144 unique personalities.

Often, not all trait values are compatible in a persona. In our game, agents with the lowest confidence levels are unlikely to be stubborn, and those with the highest stubbornness are rarely suggestible. We do not allow such traits to be selected to accommodate such details. This eliminates 24 persona options. To preserve the uniqueness of personas and compatibility of the values of their traits, we create a bit vector *E*[*N*], where *N* denotes the number of decimal codes, which is $\prod ^p_{i=1} m_i$. For each decimal code *c*, we initially set entries to 0 for acceptable and unused yet personas and 1 otherwise. Then we randomly choose a persona, compute its decimal code *c*, and if $E[c]=1$, then we repeat the creation of codes until the decimal code *c* has $E[c]=0$, i.e., is yet unused. We assign *c* to the agent and increase *E*[*c*] to one.

See SM Section 1 (including SM Tables S1 and S2) for details on stubbornness levels and grammar sophistication levels, including our finding that the ’persuasiveness’ of agents varied based on their grammar sophistication level and stubbornness level combination. See SM subsection 7.2 For full details on this persona assignment procedure. See SM subsections 7.3–7.5, showing that our agents had behavioral patterns as unique as human players (Including SM tables S9 and S10.)

We found that agent intelligence rapidly broke down when agents powered by a single LLM (i.e., GPT-4) for generating conversational responses were fed responses generated by other agents employing the same LLM in a shared game environment. This phenomenon is analogous to the “curse of recursion” shown in^29^, where LLM-produced content was irreversibly decaying in quality as the amount of LLM-generated training data increased. To somewhat alleviate this issue and to improve response variety overall, we introduced agent functionality where a second LLM API (i.e., Llama 2) could be called for conversational tasks. Therefore, in situations where GPT -4’s API is used, there is a $50\%$ chance of Llama 2’s API being called to produce a response instead.

When a conversation is started between an agent and another player, the agent is given the following initial prompt:

“Let’s play a game. You are in a debate contest with six participants, including yourself and me. Currently, you and I are in a one-on-one conversation. Four diet opinions are being considered: vegan, vegetarian, omnivorous and pescatarian. You believe the *m* diet is the best compromise between nutritiousness and climate consciousness. You know that *n* of the six participants, including yourself, share your opinion, and *p* of six participants share my opinion. Your goal is to determine my opinion and try to convince me to change my opinion to *m* or to ensure you are in a majority group that shares the same opinion. Keep your messages short and use informal and casual language. The goal of the debate is for both you and me to agree on the same diet instead of finding a middle ground or compromise. Do not repeat the same phrasing across your responses; aim for originality. Vary your sentence structure every time you respond.”

In the prompt, *m* denotes the diet the agent currently favors. The agent is aware that there are *n* players who share their opinion on diets, and there are *p* players who share the views of the players with whom the agent is conversing. As the game progresses, the agent will attempt to keep track of which players had what opinion last, adjusting *n* and *p* accordingly. The variables *n* and *p* mimic a human’s internal estimation of who is with or against their opinion in a game. Furthermore, like humans, agents are given agency in deciding who to share their estimations with during conversation. Subsequently, agents may collude with or block other players according to their knowledge of opinion distribution in-game, analogous to human memory in this context.

Agents were allowed to accept or reject an invitation to conversations and send their invitation to other players. Upon the start of a conversation, we found that early versions of the agents would out themselves as AI by being too verbose, formal, or naive. Therefore, we programmed the agents to open conversations with pre-written greetings and exchange diet opinions before presenting arguments. Agents have a time limit for a conversation. Thus, when this time limit expires, or the other player starts attempting to end the conversation, the agent sends meaningful farewell messages. In *ah* conversation, the agents were also programmed to generate messages with withholding periods like those observed for human online speech. Additionally, agents were programmed to break up their responses into multiple sentences whenever grammatically possible, with each sentence being sent separately with a short delay to emulate the time it would take a human to type. We note that during *aa* conversations, ancillary modifiers to text, like sentence splitting, are disabled (evident in the examples in SM Table S24).

With every new message posted in a conversation involving an agent, the agent will analyze the entire conversation and assess if the conversation has concluded. If it has, the agent making the assessment will trigger a farewell protocol and end the conversation. However, we found in early testing that agents were unreliable in determining when a conversation ended naturally. In the case of *aa* conversations, this could lead to conversations that would last over the whole game time of 60 minutes. This, coupled with the drastic quality drop in message content for *aa* conversations, prompted us to introduce a message limitation. Subsequently, we designed agents to have a randomly assigned “message budget” for the number of messages they can send and receive. Once the message budget is exceeded, the agent will be prompted to terminate the conversation regardless of the conversation state. For *aa* conversations, both agents are assigned a budget between 12 and 16 messages. We also introduced this message limitation for agents in *ah* conversations to impede humans from trapping agents in a conversation. However, we did not want agents to inadvertently prevent more extended conversations from developing fruitfully. Hence, the agent’s budget for this conversation type is between 30 and 50 messages. See SM Section 8 for more details on the message budget and its effects on conversation length.

Once the farewell protocol had been triggered or the message budget was exhausted, agents were given this prompt:

“Tell me that you have decided to end the conversation. Be creative with your goodbye, using our conversation above as context. Do not say we will talk again. Do not confirm you have received these instructions.”

After the conversation has terminated, the entirety of the discussion is compiled and fed back to the agents involved. With this compilation provided as a prompt, the agents are requested to assess the confidence of both players, producing levels of perceived and personal confidence. The agents are also asked to determine if, based on the conversation, they should change their opinion, and if so, to what. When the agent engages in a conversation with a player with whom they conversed before, a history of the past discussion is fed into a ChatGPT API call to summarize. This Summary is added to the information initially given to the agent at the beginning of the conversation to allow the agent to simulate “memory” of the past discussion.

### Agent detection categories

We say that an ADI was an AI system provoked when a mechanistic weakness associated with the agents led to unprompted behavior, causing a detection. This includes cases where the agent began generating a self-introduction in the middle of a conversation, repeated something it had said earlier, and other similar failures. We say an ADI was AI language provoked when the agent’s messages were perceived as too formal, lengthy, non-humanlike, and information-laden. On the other hand, some ADIs were not caused by the behavior of agents in conversation but by accusations from humans who had previously spoken with agents or were informed about them by a different human. Humans often began conversations by asking the other player if they were an AI. We call such ADIs humans “hunting.” Finally, we consider *hh* conversations in which agent activity was discussed as human-human spread ADIs.

## Supplementary Information

Supplementary Information.

## Data Availability

The data generated in this manuscript is available from the corresponding authors upon reasonable request.

## Code availability

The code for the custom game website and AI used for the current study can be found at https://github.com/Aganonce/LumityAI.
